# MinLinMo: a minimalist approach to variable selection and linear model prediction

**DOI:** 10.1186/s12859-024-06000-4

**Published:** 2024-12-18

**Authors:** Jon Bohlin, Siri E. Håberg, Per Magnus, Håkon K. Gjessing

**Affiliations:** 1 Department of Method Development and Analytics, Section for modeling and bioinformatics, Norwegian Institute of Public Health, Oslo, Norway; 2 Centre for Fertility and Health, Norwegian Institute of Public Health, Oslo, Norway; 3 Department of Global Public Health and Primary Care, University of Bergen, Bergen, Norway

**Keywords:** Variable selection, Parsimonious linear models, Machine learning, $n\ll p$ regression

## Abstract

Generating prediction models from high dimensional data often result in large models with many predictors. Causal inference for such models can therefore be difficult or even impossible in practice. The stand-alone software package MinLinMo emphasizes small linear prediction models over highest possible predictability with a particular focus on including variables correlated with the outcome, minimal memory usage and speed. MinLinMo is demonstrated on large epigenetic datasets with prediction models for chronological age, gestational age, and birth weight comprising, respectively, 15, 14 and 10 predictors. The parsimonious MinLinMo models perform comparably to established prediction models requiring hundreds of predictors.

**Supplementary Information:**

The online version contains supplementary material available at 10.1186/s12859-024-06000-4.

## Background

Machine- and statistical learning models are trained on data to predict an outcome on test data [1]. As opposed to classical statistical inference, the purpose is not to assess the validity of each explanatory variable in the model but to produce predictions with superior generalization to novel or unseen data [2]. A successful model will thus generalize well to datasets, often referred to as test- or validation datasets, that are independent of the data the models have been trained on. Models that fit the training data well but do not generalize to novel data are said to be over-fitted [1]. Since prediction models focus on predictive ability, the variables emphasized by these models, in one way or another, tend to be reflective of that. When prediction models contain many variables, interpretation can be difficult and therefore such models are sometimes referred to as “black boxes” [2].

There exists several methods for selecting variables from datasets with more samples than predictors ($n\ge p$). The most familiar examples of such methods include best subset selection, forward- and backward selection as well as step-wise selection [3]. The best subset selection method tries to find a subset of predictors that maximize prediction accuracy. Forward selection is based on adding predictors iteratively as long as the model’s goodness-of-fit, often measured by the coefficient of variation $R^2$ for linear models, increases. Backward selection is the opposite of forward selection; all predictors are added to the model and those that do not increase the model’s goodness-of-fit statistic are iteratively removed. Step-wise selection, often used with best subset selection, is based on adding or removing predictors for the sake of increasing goodness-of-fit.

For datasets with a large number of predictors *p* compared to the number of samples *n*, often referred to as $n\ll p$ datasets, backward selection is not possible as linear regression requires that the number of samples in a model is at least as large as the number of variables [3]. Best subset, step-wise- and forward selection methods, on the other hand, can potentially return a result if the number of predictors correlating with the outcome is lower than the number of samples. For datasets with many predictors, these methods can be inefficient since the number of regression models will equal the number of predictors tested.

Datasets with $n\ll p$ have far fewer methods for variable selection [4]. The most commonly used method in such circumstances is likely the Elastic Net, which allows for a wide range of different modeling options [5]. An Elastic Net model requires the tuning of two parameters ($\alpha$ and $\lambda$) that affect the number variables to be included in the prediction models (see [6] for an introduction to the Elastic Net models). The $\lambda$ parameter is tuned, using leave-one-out cross-validation, so that the estimated model coefficients result in a prediction model with the lowest mean square error (MSE). This is typically known as the minimum MSE penalty. It has been shown that allowing for a penalty within one standard error (1SE) of the minimum MSE, estimated during cross-validation, does not change prediction accuracy much but the number of selected variables can drop dramatically. This phenomenon has been termed the “The one standard-error rule” (1SE rule) [3]. The $\alpha$ parameter, which decides the type of penalty the Elastic Net employs, takes values between 0 and 1 with 0 being the least parsimonious models, which includes all explanatory variables (known as Ridge regression), and 1 the most (the Lasso). The Lasso [7], based on a variant of best subset selection, typically selects fewer variables than other Elastic Net variants but manages occasionally to exhibit comparable predictive accuracy [3]. As such, the Lasso is not only an attractive model for prediction, when the number of predictors is high and the number of samples low, but also for variable selection. As genetic- and epigenetic datasets often contain hundreds of thousands of explanatory variables [8] even prediction models produced by the Lasso may contain hundreds or even thousands of explanatory variables making interpretation of the selected variables overwhelming.

A procedure known as Stability selection [4] can refine the number of variables selected from Elastic Net regressions. This is typically done by multiple rounds of Elastic Net regressions on random sub-samples of the dataset, not unlike bootstrapping, where the retained predictors are those being repeatedly selected during subsequent rounds [4]. Although Stability selection is able to reduce the number of predictors dramatically and produce consistent results while maintaining acceptable prediction performance to the Elastic Net [9], the method is somewhat involved and extremely time consuming, especially for large datasets, due to the many rounds of Elastic Net regressions required.

The nature of the association between the explanatory variables and the outcome can vary. In some cases, many explanatory variables that correlate weakly with the outcome may result in strong prediction models [3]. Such models are often best handled using “boosting” methods [1, 3]. In other instances, the situation can be reversed; effective prediction models can emerge from utilizing only a handful of explanatory variables. A common scenario however is prediction models with a mixture of the two examples, i.e. models with some explanatory variables correlating strongly with the outcome but with the majority of the variables correlating weakly. Explanatory variables may correlate weakly due to an indirect relation to the outcome, by chance (false positives) or simply because they have a weak effect on the outcome. Since genetic- and epigenetic array data often only contain a subset of all possible sites (i.e. SNPs or CpGs, respectively) [10], these could potentially still be associated with a site not present on the array [8]. Moreover, in datasets with hundreds of thousands of variables many will be included by a prediction model simply by chance and these could be difficult to separate from variables that correlate weakly with the outcome for one reason or another. Hence, predictive models that aim at increasing accuracy may result in models with reduced interpretability simply due to the overwhelming number of variables selected [1].

Here, the stand-alone tool MinLinMo is introduced for linear $n\ll p$ models which seeks to remedy some of the above mentioned limitations. MinLinMo rejects spurious, low correlating variables in favor of parsimonious models while concurrently achieving acceptable prediction accuracy to larger models produced by methods such as the Elastic Net. The proposed method is demonstrated on real epigenetic datasets where it extracts far fewer predictors at a fraction of the time and memory consumption than the ‘glmnet’ package [11], which estimates the Elastic Net models in the statistical environment R [12]. Although no variable selector with a similar aim to the one presented here is known to the authors, MinLinMo is, somewhat unfairly, compared to the Elastic Net which aims at generating the most accurate prediction models, regardless of model size.

Three models are presented based on DNA methylation (DNAm) data from the Illumina Infinium MethylationEPIC platform [8]: adult age- and gestational age predictors (often referred to as “clocks”) as well as a birth weight predictor. These three predictors are made using both MinLinMo and the Lasso from the Elastic Net. Two types of penalties are considered for the Lasso, one based on the minimum MSE penalty and another based on the 1SE rule penalty. The former penalty emphasize prediction accuracy, the latter parsimonious prediction models. The MinLinMo age predictor is compared to both the Lasso predictors as well as other established epigenetic aging clocks. A comparison of the MinLinMo-based gestational age predictor with regards to a similar clock based on Stability selection [9] is also provided.

## Implementation

### A simple overview of MinLinMo

**Fig. 1:**
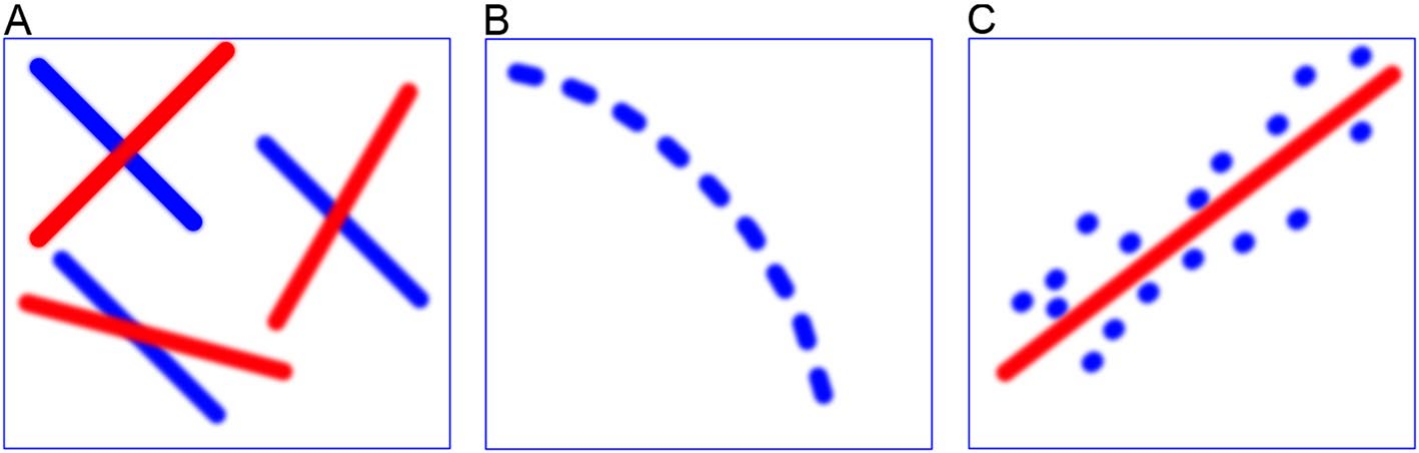
**A** MinLinMo first performs Pearson correlations between the outcome and all the predictors in the dataset. **B** The indices of the predictors that correlate with the outcome are subsequently submitted to a priority queue with an emphasis on the correlation coefficient. **C** A prediction model is constructed by popping from the queue progressively less correlating predictors that are regressed against the outcome and kept in the model if the adjusted $R^2$ increases

As can be seen from Fig. 1, MinLinMo uses several phases to select variables. First, the dataset, meaning the outcome vector and the predictor matrix, is loaded into random access memory (RAM). It must be assumed that the association between the outcome and the predictors is linear. The outcome and predictor files given to MinLinMo must be comma separated (.csv files) with headers and there can be no missing values or columns of different length (see also Additional file 1 or https://github.com/JonBohlin/MinLinMo). Row names must also be removed but column names are required, also for the file containing the outcome vector. Categorical variables must be converted to indicators (i.e. 0/1).

After the dataset is loaded into memory, several pointers are made to the predictor matrix, corresponding to the number of threads available, so that only one copy resides in memory at all times. The first step in the analysis is to perform Pearson correlations on each column in the prediction matrix against the outcome. To speed this process up, multiple threads are used together with SIMD (Single Instruction Multiple Data) technology present on most modern computers. Threads were used to allow for data sharing. Hence, each thread reads from an isolated part of the dataset instead of copying the data into memory as would be necessary when using processes. The Pearson correlation step typically uses only a few seconds, regardless of dataset size, on most recent computers.

After the Pearson correlation step, indices to the explanatory variables with the absolute value of a Pearson correlation coefficient above a given threshold, set to 0.1 by default, are added to a priority queue. In practice, indices to the variables are added one by one during estimation to a dynamic list and sorted in descending order with respect to the correlation coefficients after all variables are tested. This is done to reduce overhead during estimation. Based on the priority queue, a linear regression model, estimated using QR decomposition, is created starting with the highest correlating explanatory variable with the outcome. Subsequent explanatory variables are popped from the priority queue and tested against the regression model residuals (i.e. the model subtracted from the outcome) one by one using the absolute value of Pearson correlation. If the correlation between a predictor and model residuals is above a given threshold (again 0.1 by default) a QR decomposition is performed together with the appropriate parameter estimations. If the adjusted $R^2$, taking into account the degrees of freedom of the current model, increases more than the given threshold, which is set to $R^2 = 0.01$ by default, the predictor is included in the final model. This step penalizes large models and rejects spuriously correlating variables while time is saved by not performing QR decomposition if predictors from the priority queue correlates below the given threshold with the residuals.

After all the variables in the priority queue have been tested, MinLinMo outputs the associated variables of the final model together with the estimated intercept and slopes that can be used for prediction. As default, each included predictor must improve the model with 1% (i.e., $\Delta R^2 \ge 0.01$), but both initial correlation-, residual correlation and $R^2$ thresholds can be changed by the user. Since the model building part of MinLinMo can not be parallelized, reducing the residual Pearson correlation threshold (and to some extent the $R^2$ threshold) may increase compute time. Additional technical details can be found below.

**Fig. 2:**
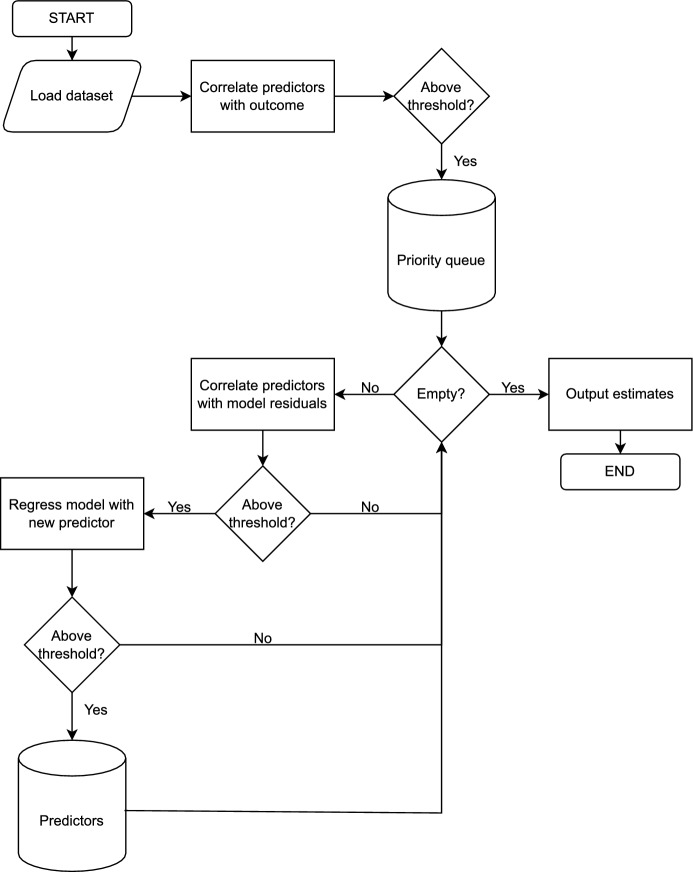
The MinLinMo algorithm. The predictor matrix and outcome vector are loaded into memory. Pearson correlations are subsequently performed between the predictors and the outcome. The predictors correlating above a given threshold (0.1 by default) are submitted to a priority queue. The first predictor popped from the priority queue is regressed against the outcome. Eventual predictors popped from the priority queue are correlated against the current prediction model residuals. If the correlation is above a given value (0.1 by default) the predictor is included in the prediction model. If the model’s goodness-of-fit statistic $R^2$ improves by more than a defined value (0.01 by default) the predictor is added to the set of final model predictors. Otherwise the predictor is discarded and a new predictor is popped from the priority queue starting the procedure over again from there on. This continues until the priority queue is empty after which MinLinMo outputs the selected predictors, as well as their corresponding estimates, and quits

### Technical details

The program was implemented in C++ version 14 on Linux, OS X (ARM/Apple Silicon) and Windows platforms (a compiled version for Windows is available at both https://github.com/JonBohlin/MinLinMo and https://zenodo.org/records/10149465 together with all necessary libraries). The source code is available on GitHub: https://github.com/JonBohlin/MinLinMo and can be downloaded and compiled using GCC, Clang and CL for the three platforms, respectively. Detailed instructions on how compilation can be performed on all platforms can be found there as well as in Additional file 1.

MinLinMo is currently dependent on the GNU Scientific Library version 2.7 (GSL) for QR-decomposition-based model building. The GSL libary, in turn, depends on a BLAS-type library (Basic Linear Algebra Subprograms) that is needed for the linear algebra part. Most installations of GSL includes a BLAS-type library that may not be optimal in terms of speed. The QR decomposition from the GSL library [13, 14] is based on the state-of-the-art Householder algorithm [15]. A complete overview of the MinLinMo algorithm is shown in Fig. 2.

The Pearson correlation phase in MinLinMo is straightforwardly implemented but requires SIMD technology, which is present on most modern computers. However, since SIMD libraries are not standardized, adapting MinLinMo to platforms other than those currently supported may require changes to the code.

MinLinMo was trained on the Norwegian Mother, Father and Child Birth Cohort (MoBa) [16] datasets discussed below. The selected variables and their corresponding estimates provided by MinLinMo were subsequently used to obtain predicted- age, gestational age and birth weight on the test datasets that are also described below in more detail. It should be noted that since MinLinMo is based on QR decomposition its model estimates are equal to those from a standard linear regression. Hence, regressing the outcome on the MinLinMo selected variables will give the same coefficient estimates as running MinLinMo. Due to implicit multiple testing issues *p* values for the training dataset will not be valid, however.

### The elastic net models

Since the point of this study was to showcase MinLinMo and parsimonious prediction models, the Lasso was selected from the Elastic Net, i.e. $\alpha$ parameter set to 1. The Lasso selects a minimal subset of predictors and can, under certain conditions, yield models that achieve optimal prediction accuracy [3]. For comparison, the Lasso models were trained and tested on exactly the same datasets as MinLinMo. Cross-validation was employed to obtain estimates of the penalty parameter $\lambda$ that affects coefficient estimates. To compare the Elastic Net models justly with MinLinMo in terms of speed, the ‘doMC’ package [17] was used to enable parallelization. However, the substantial memory requirements of parallelizing the Elastic Net trained on the large DNAm datasets did not allow for any major speedup.

The Elastic Net procedure is optimized towards better predictions regardless of the number of predictors included in the models. The number of predictors can be reduced substantially by employing the 1SE rule penalty, as discussed above, however the effect on prediction accuracy is not known for the outcomes tested here. Hence, the differences in prediction accuracy with respect to the minimum and the 1SE rule penalties were also explored. Nevertheless, the comparison between MinLinMo and Elastic Net is not entirely fair since the main goal of the Elastic Net is prediction accuracy and not parsimonious models.

### Comparison of the different prediction models

For the different (gestational) age clocks, the estimated coefficients were obtained from the respective publications [9, 18–20] and used, together with the provided CpG identifiers, on the test datasets to calculate the corresponding predicted outcomes. Several of the CpGs were missing on the test dataset GSE116339 for all of the age clocks, except the MinLinMo-based clock, which could have affected their performance.

To compare the different prediction models for age- and gestational age as well as birth weight against each other, the given outcome (i.e. age, gestational age and birth weight) on the test dataset was regressed against the respective predicted outcome, from one of the above mentioned predictors/clocks, using standard linear regression. These regression models also ensured that the predictive outcomes for age, gestational age and birth weight were all normalized to a uniform scale (years/months, days and grams, respectively). The resulting adjusted $R^2$ from each model, i.e. the variance explained taking model size into consideration, was then used to assess the accuracy of the predicted models.

The Four CpG Clock (FCC), Eight CpG Clock (ECC) and reduced birth weight predictor were all based on the respective MinLinMo selected variables. The given outcomes for age, gestational age and birth weight on the respective test datasets were regressed against the MinLinMo selected variables, one by one, also on the test datasets, starting with the first and continuing as long the regression models improved with respect to $R^2$ (see Additional file 2).

The variance of the residuals from the regression analyses described above were subsequently compared using Fisher’s F test [21] as an additional examination of predictive accuracy. By employing the F test on the regression residuals, an assessment could be made regarding whether the predictive accuracy between two methods (e.g. MinLinMo and Lasso) was significantly different ($$p < 0.05)$$ or not ($$p > 0.05$$). However, due to considerable correlations between the predicted values from the different methods, as well as the dependence structures present in the DNAm datasets [22], a standard F test will likely not provide accurate *p* values. To remedy this, the regression models with given age, gestational age and birth weight as outcomes and corresponding predicted values, for each method (e.g. MinLinMo, Lasso, etc.), were bootstrapped 1,000 times. More specifically, comparison between two predictors was performed by simultaneously bootstrapping both linear regression models with a given outcome against the predicted values for both methods [23] (e.g. age models for MinLinMo and the Lasso Min model). For each bootstrap replicate, an F test is performed on the variance of both models’ residuals and the resulting F statistic is recorded. The F statistics, recorded during bootstrapping, are subsequently used to estimate the mean F statistic for each method and predicted outcome as well as to obtain *p* values, which were calculated as follows:

Let $F_{lower}$ and $F_{upper}$ designate the 95% confidence intervals for the *F* statistic. These values can be obtained by passing the values 0.05 and 0.95, respectively for $F_{lower}$ and $F_{upper}$, together with degrees of freedom (DoF) for each regression model, to a cumulative distribution function for the F distribution. Since the regression models comparing outcome and predicted values only have one predictor, the DoF can be set to the number of predicted values on the test dataset - 2: 676 for the age predictors and 436 for the gestational age and birth weight predictors. $F_{lower}$ and $F_{upper}$ can, for instance, be obtained in R with the function calls **qf(0.05, DoF, DoF)** and **qf(0.95, DoF, DoF)**, respectively. When the $F_{lower}$ and $F_{upper}$ values are obtained, the *p* values can be computed:

$$\begin{aligned} p=1-\frac{\#(F<F_{lower})+\#(F>F_{upper})}{\text {The total number of bootstraps}} \end{aligned}$$

$\#(F<F_{lower})+\#(F>F_{upper})$ designates the addition of the number of sampled F statistics above $F_{upper}$ and below $F_{lower}$.

All statistical procedures were performed with the statistical package R version 4.1.2 [12] and MinLinMo v.1.0. The figures were also created with R except for the flow chart which was created with *draw.io* version 24.2.3 (https://app.diagrams.net/).

### Description of the datasets used for training and testing

The datasets used for training were all taken from the Norwegian Mother, Father and Child Cohort Study (MoBa) [16]. The EPIC v.1.0 dataset used to train the birth weight and gestational age models was based on cord blood and the same used in the Stability selection-based clock study [9]. The initial quality control (QC) procedures were identical. Some differences with regards to sampling can be noted though; the dataset used in the present study consisted of 2,188 samples in total (51.5% females and 48.5% males). The two independent samplings from MoBa consisted of 988 + 1,207 random control samples = 2,197 samples in total including 9 samples with missing gestational age that were removed. The number of CpGs was identical to the previous study with 769,139 CpGs in total as all missing and non-overlapping CpGs were removed during QC [24]. The two MoBa datasets were randomly combined into a training dataset with 1750 samples (80%, 51.1% females, 48.4% males) and a test dataset with 438 samples (20%, 51.1% females, 48.9% males). Due to the random mixing, both training- and test datasets consisted of a random mixture of samples from both MoBa datasets. The median gestational age in total was 281 days, the same for training, and 281.5 for testing. Gestational age was estimated from ultrasound measurements. The average birth weight was 3625.7 g for training, 3666.6 g for testing and 3633.9 g for the total sample. The dataset used for training the chronological age prediction models was also from MoBa. It was based on whole blood and consisted of 1966 adults (50.1 % females and 49.9% males). The median age for the adults was 11,384 days (31.2 years) in total. The QC has been described previously [24] but a broad overview is included in Additional file 3.

The dataset used for testing the chronological age models was downloaded from the Gene Expression Omnibus web site https://www.ncbi.nlm.nih.gov/geo/, accession number GSE116339. This dataset was based on the EPIC v.1 platform and quality assessments and controls were already performed. It was taken from a study assessing the effects of polybrominated biphenyl (PBB) exposure on DNAm in blood [25]. The study participants were 679 in total, 398 females (59 %) and 280 males (41 %). The mean age of the participants was 53.9 years (19,671 days). All CpGs with missing values were removed leaving 763,795 CpGs for analysis (see also Additional file 3).

The random dataset used to assess false positives was created simply by sampling 500,000 (columns) * 1000 (rows) times from a Gaussian distribution with mean 0 and standard deviation 1. The outcome was randomly sampled 1000 times from a Gaussian distribution, also with mean 0 and standard deviation 1 (see also Additional file 1 and examples there).

## Results

### Evaluation of MinLinMo

To evaluate MinLinMo’s performance we created three prediction models from large ($n\ll p$) DNAm datasets (see the Implementation section for more information regarding the datasets). The three prediction models were for adult- and gestational age in addition to one for newborns’ birth weight. We also created Lasso-based prediction models for the same three outcomes so MinLinMo could be compared against a state-of-the-art method [26]. Moreover, the MinLinMo gestational age clock was compared to a recent gestational age clock [9], based on the Stability selection method [4], which was made in order to reduce the number of predictors as much as possible. All prediction models were also tested on a randomly generated dataset to assess the ability of the models to reject false positive predictors.

### Aging clocks

Both MinLinMo and Lasso based age prediction models were trained on the Norwegian Mother, Father and Child Cohort Study dataset (MoBa, see the Implementation section for more details) consisting of 1966 samples and 770,586 predictors (i.e. DNAm sites also known as CpGs) taken from peripheral blood. The prediction models were tested on an independent dataset (GSE116339), containing 678 samples and 763,795 predictors, also based on blood, and compared to other established DNAm-based age predictors. These included Steve Horvath’s 353 CpG pan-tissue clock [19], Horvath’s state-of-the-art 421 CpG Skin and Blood clock [20], Hannum’s 71 CpG whole blood clock [18] as well as two MoBa-trained Lasso predictors: one, which selected 1235 CpGs, was based on the minimum MSE penalty (the Lasso Min clock) while the other predictor was based on the 1SE rule penalty (the Lasso 1SE clock) extracting 421 CpGs.

Some of the clock CpGs were missing on the test dataset so that the same clocks respectively consisted of 322 CpGs (pan-tissue clock), 376 CpGs (Skin and blood clock), 61 CpGs (Hannum’s whole blood clock), 1170 (Lasso Min clock) and 398 (Lasso 1SE clock). The MinLinMo clock was based on 15 CpGs, all of which were present on the test dataset. See Additional file 4 for more information regarding the CpGs in the different clocks.

**Fig. 3:**
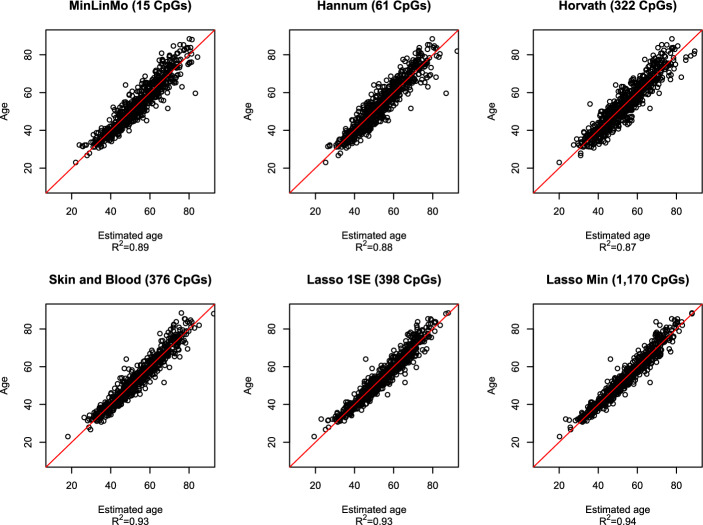
Comparison of selected epigenetic aging predictors. The vertical axis designates given age in years while the horizontal axis represents predicted age. The red line is from the regression model with given age as outcome and predicted age as the explanatory variable, for each respective clock

To compare the predictive performance between the different epigenetic clocks, chronological age (outcome) was regressed on estimated age (predictor). This ensured that all predictors were on a similar scale (i.e. years). As can be seen from Fig. 3 and Additional file 2, the highest goodness-of-fit statistic $R^2$, between given- and predicted age, on the test dataset (GSE116339) was for the Lasso Min clock ($R^2=0.94$), followed by the Lasso 1SE and Horvath’s Skin and blood clock ($R^2=0.93$). For the remaining clocks, MinLinMo (15 CpGs) obtained an $R^2$ of 0.89, followed by the Hannum’s clock (61 CpGs, $R^2=0.88$) and, lastly, Horvath’s pan-tissue clock (322 CpGs, $R^2=0.87$).

MinLinMo was additionally tested with the adjusted model parameters 0.05, 0.005 and 0.05 for initial Pearson correlations, $\Delta R^2$ and residual correlation, respectively. The model selected 27 CpGs and obtained an $R^2=0.9$ on the test dataset, as compared with 15 CpGs and $R^2 =0.89$ for the default parameter settings, and was therefore not pursued further.

Since the $R^2$ statistic is a somewhat rough estimate for predictive ability, a bootstrapped variance test (based on Fisher’s F test, see the Implementation section for details) was performed between the residuals from the regression models [23], having age as the outcome and predicated age as the explanatory variable, for each epigenetic clock. Significantly smaller (i.e. $p<0.05$ ) residual variance indicates superior prediction accuracy (see the Implementation section and Additional files 2 and 5 for more details). The variance of the residuals from the MinLinMo-based age predictor was comparable to both the Hannum clock ($p=0.90$) and Horvath clock ($p=0.54$). The Hannun- and Horvath clock residuals were also of comparable variance ($p=0.67$). The clocks with the significantly lowest residual variance were the Lasso-based clocks and the Skin and blood clock. There was no significant residual variance difference between the Lasso Min clock and the Skin and blood clock ($p=0.5$), there was also no significant variance difference between the Lasso Min and Lasso 1SE clock residuals ($p=0.54$). Neither was there any significant variance difference between the residuals from the Skin and blood clock and the Lasso 1SE clock ($p=0.86$), suggesting the differences between the Lasso clocks and the Skin and blood clock are negligible. The variance of the residuals from the Lasso clocks and the Skin and blood clock were all significantly lower than for the MinLinMo-, Hannum- and Horvath clocks ($p < 0.001$, in all instances.). See Additional file 2 for statistical details regarding these comparisons.

The MinLinMo selected aging clock consisted of 15 CpGs. A further scrutiny of these CpGs indicated that only four constituted an aging clock obtaining an $R^2 = 0.87$ (as compared to the full MinLinMo clock of $R^2=0.89$) and thus explaining the majority of the observed variance. The residual variance of the Four CpG clock (FCC) was not significantly different from either the full MinLinMo clock ($p=0.14$), the Hannum clock ($p=0.31$) or the pan-tissue clock ($p=0.76$) on the test dataset. The CpGs constituting the FCC were primarily associated with the two known aging genes *ELOLV2* and *KLF14*, linked to metabolism [27, 28], which were hypermethylated, followed by a tumour suppressing gene *RNF180* [29], and *DYTN*, a gene with unknown function, that were both hypomethylated. All CpGs related to these genes were located in the promoter region except for the CpG linked to the *DYTN* gene, which was located in the gene body. More information regarding these CpGs and their corresponding genes can be found in Additional file 4 while Additional file 2 contains more detailed results regarding the statistical comparisons.

**Fig. 4:**
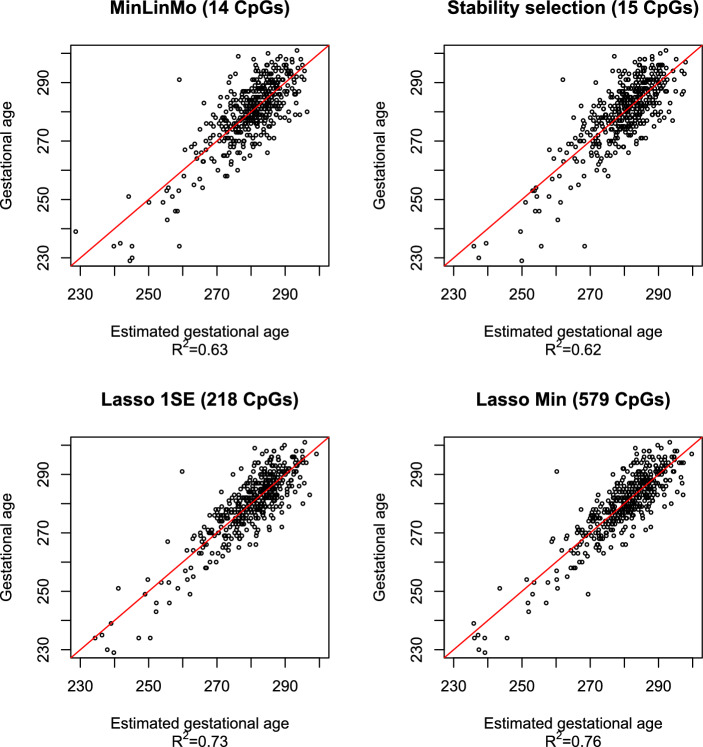
Comparison between epigenetic gestational age predictors. The vertical axis designates given gestational age in days while the horizontal axis represents predicted gestational age. The red line is the regression model line

### The gestational age clock

The MinLinMo prediction model for gestational age selected 14 predictors in total obtaining an $R^2=0.63$ between given and predicted gestational age on the test dataset (see Additional file 2). This result was slightly above the 15 explanatory variables obtained previously using Stability selection [9], namely $R^2=0.62$ (see Fig. 4). A bootstrapped variance test, as described above (see also the Implementation section for details), was also performed on the residuals, from given gestational age regressed on predicted, where no significant difference was found between the two clocks ($p=0.95$). 10 CpGs were common to both clocks (see Additional file 6, which also includes overlapping CpGs with other clocks).

The Lasso-based clocks performed better than both the Stability selection- and MinLinMo based models. Both in terms of $R^2$ ($R^2=0.76$ and $R^2=0.73$ for the Lasso Min and Lasso 1SE models, respectively) and the variance test on the residuals from gestational age (see Additional File 5) regressed on predicted gestational age ($p<0.001$). There was no significant difference with respect to residual variability between the Lasso 1SE and Lasso Min models ($p=0.97$).

The number of CpGs selected by the Elastic Net algorithm for gestational age (579 for Lasso Min and 218 for Lasso 1SE) were substantially larger than for the Stability selection- (15 CpGs) and MinLinMo (14 CpGs) models. 10 CpGs were found to be overlapping with MinLinMo and Stability selection models, for both types of penalty estimations (i.e. minimum penalty and 1SE rule penalty). Details regarding the CpGs included in all the gestational age prediction models discussed here can be found in Additional file 7.

Further scrutiny of the MinLinMo selected CpGs revealed that only 8 CpGs were needed to obtain a prediction model with $R^2=0.62$. The residual variance for gestational age regressed on the outcome of this Eight CpG clock (ECC) was comparable to the full MinLinMo clock ($p=0.99$). The CpGs of the ECC were found to be close to the genes *NCOR2*, *SETBP1*, *HLA-DMB*, *EFR3B* which were all hypomethylated, and to *ATP6V0A4*, *IGF2BP1*, *SRC* and *LRBA* which were hypermethylated. The CpGs linked to these genes were located in the gene body except for the CpGs close to *NCOR2* and *ATP6V0A4* which were both located in the promoter region. The genes *NCOR2*, *IGF2BP1*, *SETBP1*, *SRC* were all associated with development [9, 30–32]. Both *LRBA* and *HLA-DMB* genes were linked to the immune system [33, 34] while *EFR3B* was associated with signaling [35]. The *ATP6V0A4* gene codes for an ATP-dependent proton pump [36].

**Fig. 5:**
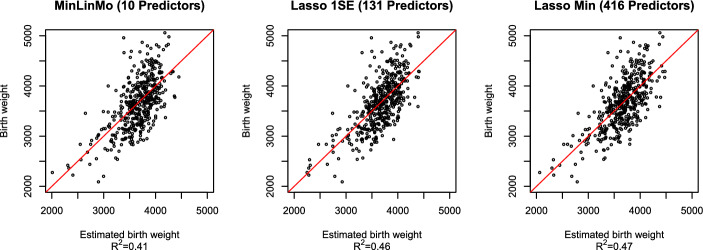
Comparison between epigenetic birth weight predictors. The vertical axis designates given birth weight in grams while the horizontal axis represents predicted birth weight. The designated predictors for all models consist of CpGs + gestational age. The red line represents the regression line for each model

### The birth weight predictor

Gestational age was introduced as an explanatory variable for the epigenetic birth weight prediction model, together with all 769,139 CpGs. Gestational age was selected by both the Elastic Net- and MinLinMo models. For this predictor, the accuracy between the Elastic Net and MinLinMo was closer but slightly better for the Elastic Net in terms of $R^2$, i.e. $R^2=0.47$ and $R^2=0.46$ for the Lasso Min and 1SE models respectively, compared with $R^2=0.41$ for MinLinMo (see Fig. 5 and Additional file 2). Comparing the variance of the residuals from regressing birth weight on predicted birth weight from the different predictors (see Additional file 5 and the Implementation section for more details), there was no significant difference between Lasso Min and Lasso 1SE ($p=0.99$) as well as between MinLinMo and the Lasso models ($p=0.9$ and $p=0.99$, for the Lasso Min and 1SE models, respectively).

The number of predictors selected by the Lasso was again orders of magnitude larger than MinLinMo, which only needed 9 CpGs and gestational age. The Lasso predictors selected 416 and 131 respectively for the Min and 1SE models, where one of the predictors was gestational age for both models.

5 predictors, including gestational age, were found to overlap between the methods (see Additional file 6). 1 CpG selected by MinLinMo was found to overlap with the 19 CpGs identified in an early study on DNAm and birth weight [37]. In a recent meta-analysis also exploring the association between birth weight and DNAm [38], 3 of MinLinMo’s 9 CpGs were found to overlap with the reported 8,170 CpGs. While the remaining CpGs selected by MinLinMo were specific for the EPIC platform, one CpG was not (cg00294109).

A further scrutiny of the MinLinMo-based birth weight predictor revealed that 3 CpGs and gestational age (see Additional file 2) resulted in a prediction model with comparable accuracy to the full MinLinMo prediction model ($R^2=0.4$ as compared to $R^2=0.41$). Regressing birth weight on predicted birth weight for both the full MinLinMo model and the reduced model revealed no statistical difference with regards to residual variance ($p=0.92$).

The strongest predictor for birth weight was found to be gestational age, followed by CpGs related to the genes *NEDDL4*, *LOC400867* and *FOXO3*. There was a positive association between birth weight and gestational age as well as the CpG representing the *FOXO3* gene (*i.e*. it was hypermethylated.). The CpGs representing the *NEDDL4* and *LOC400867* genes were both negatively associated with birth weight (i.e. they were hypomethylated). All CpGs were located in the gene bodies of their respective genes. All genes, *NEDDL4* [39], *LOC400867* [40] and *FOXO3* [41] appear to be involved in different aspects related to metabolism. See Additional file 8 for details regarding the CpGs related to birth weight prediction and their corresponding gene and genomic regions.

### Simulated data

To assess how MinLinMo compares against the Lasso in terms of false positives, a random dataset was created consisting of 500,000 columns and 1,000 rows (See the Implementation section and Additional file 1 for more details). The purpose was to assess how MinLinMo compared against the state-of-the-art software in rejecting false positive explanatory variables. At default settings, we found that MinLinMo picked out 32 variables against a random outcome. The Lasso selected 117 variables when the $\lambda$ parameter was set to minimum MSE. However, when the 1SE rule was applied no predictors were selected.

**Table 1:** Time, in seconds, taken to train the MinLinMo and Lasso prediction models on the same cluster

Model	MinLinMo training time	Lasso training time
Age	44	3, 032
Gestational age	12	2, 309
Birth weight	10	2, 228
Simulated dataset	2	558

### Performance

The MinLinMo algorithm was substantially faster than the Lasso, as can be seen from Table 1. Moreover, in terms of memory usage, MinLinMo only needed the size of the dataset, in addition to the size of the prediction model, which is typically small, and some negligible scratch space. When the Lasso is run with multi-threading enabled, the dataset is multiplied in memory according to the number of threads used. Stability selection, which is the method that MinLinMo tries to emulate, is based on continuous refinements of multiple Lasso/Elastic Net runs, and is therefore as many times slower than the Lasso/Elastic Net.

## Discussion

### The inner workings of MinLinMo

MinLinMo is a tool that aims at selecting variables for small linear prediction models, typically several times smaller than the Elastic Net, while still obtaining comparable, or at least acceptable, prediction accuracy. This is obtained by carefully selecting the explanatory variables included in the final model while excluding false positives and those that only contribute marginally, below a set threshold. Furthermore, conducting a Pearson correlation analysis on all explanatory variables against the outcome and adding those with correlations above a certain threshold to a priority queue provides an efficient method to reduce the number of variables that show weak or indirect correlations. This approach ensures that the priority queue emphasizes explanatory variables with stronger correlations to the outcome. Since the previously tested variables always correlate more strongly with the outcome, subsequent variables tested will be ignored if the variance explained by the model does not improve. During prediction model construction, only the predictors that improve the model above a given adjusted $R^2$ threshold are kept, implying that the inclusion of highly correlating variables is reduced substantially. Moreover, by using adjusted $R^2$ instead of standard $R^2$ (coefficient of determination) large models are penalized. Hence, when all explanatory variables are tested only those that improve prediction accuracy and do not fully correlate with other predictors are kept. It is likely this step that allows MinLinMo to produce considerably smaller models than the Elastic Net yet still perform acceptably.

Substantial speed up is also achieved by first correlating each predictor against the current model residuals performing QR decomposition only if this correlation is above the given threshold.

### The differences between MinLinMo and the elastic net

As mentioned above, in the first phase MinLinMo tests explanatory variables directly against the outcome using Pearson correlation. In the next phase, the variables correlating with the outcome are subsequently correlated against the previous model’s residuals. Since the subsequent variables popped from the priority queue correlate less with the outcome than the previous variables, it is more likely that subsequent variables will be uncorrelated with the model residuals. If the residuals correlate above a given threshold (0.1 by default) a full regression model (i.e. QR decomposition), which is costly in terms of time, is computed. The predictor will only be included in the final model if the goodness-of-fit statistic is above a given threshold (the default requirement, which can be adjusted, is that variance explained increases at least by 1%).

The Elastic Net on the other hand, exclusively tests each explanatory variable against the model residuals, one-by-one. Those variables that correlate with the residuals are placed in an “active set”. The coefficients of the predictors in the active set are constantly adjusted according to the assumption that each added predictor correlates equally with the residuals. Excepting Ridge regression, the predictors whose coefficients are shrunk to zero are excluded from the active set but can be included later again.

One of the strengths of the Elastic Net is that multiple predictors are estimated simultaneously. The Elastic Net models can therefore potentially detect complex interaction patterns between predictors and the outcome. While MinLinMo tries to mitigate this by always fitting models whose predictors are progressively correlating less with the outcome, complex correlation structures will nevertheless likely not be detected. MinLinMo’s inferior ability to detect complex correlation structures among predictors could, on the other hand, contribute to its parsimonious models. While Elastic Net models are focused on the total correlation of a group of variables with the outcome, emphasizing more complex models with regards to the correlation structure of the predictors, MinLinMo puts more effort into detecting single predictors that correlate with the outcome. This may, however, also contribute to MinLinMo’s ability to produce small prediction models. As such, the strengths of both the Elastic Net and MinLinMo may also be their weaknesses, depending on the perspective, and therefore the models should be considered as complimentary. As far as we know, excepting models for specific outcomes [42, 43] and the Stability selection procedure [4], there are no tools directly comparable to MinLinMo’s implementation.

### The one standard error rule

We found that using the 1SE rule penalty for the Lasso models did not reduce prediction accuracy considerably, as compared to the minimum MSE penalty. However, the number of selected predictors dropped dramatically; from 1,235 to 421 CpGs for the adult age clocks, from 579 to 218 CpGs for the gestational age clocks and from 416 to 131 CpGs for the birth weight predictor. Hence, a substantial drop in selected variables can potentially be achieved with the Lasso/Elastic Net, by opting for the 1SE rule penalty instead of the minimum MSE penalty, with negligible effects on prediction accuracy.

### Stability selection

In a previous study [9] Stability selection [4] was employed, together with the Lasso, to create a minimal gestational age prediction model so that the implicated CpGs, together with their associated genes, could help elucidate the mechanisms behind epigenetic gestational age clocks [9]. Although Stability selection, as employed in that study, did manage to reduce the number of associated CpG sites, thereby producing a model comparable to what MinLinMo has demonstrated here, the tool itself was both complicated and extremely time consuming. MinLinMo was designed to provide small prediction models fast and, as mentioned above, there was no significant difference ($p=0.95$) in terms of prediction accuracy between the similarly sized MinLinMo- and the Stability selection-based clocks.

### Efficiency

MinLinMo was also built with a focus on efficiency so that it can be run on a laptop or desktop computer, even with a large $n\ll p$ dataset. MinLinMo is based on threads and these can access the same shared memory meaning that there is no need to make multiple copies in memory of the dataset, or the part that is being operated on; everything is performed using pointers to different blocks of the dataset at the same time. Our findings seem to suggest that although the ‘glmnet’ package uses the ‘doMC’ library for parallelization [17] it does not employ threads, or at least the ability of threads to share the same memory, during leave-one-out cross-validation (at least up to R version 4.1.2). If glmnet forks processes, that do not have access to other processes’ memory, it has no choice but to copy the entire dataset into memory for at least as many processes as is being forked. Regardless of what strategy glmnet uses for parallelization it seems extremely memory inefficient. However, it’s not clear whether this restriction is due to the R environment or the inner workings of the glmnet package.

### The MinLinMo-based prediction models for age, gestational age and birth weight

We have shown that compared to the Lasso, MinLinMo’s prediction accuracy is inferior. However, the number of extracted predictors were orders of magnitude smaller for MinLinMo than for the Lasso. Furthermore, in spite of the far fewer predictors selected by MinLinMo it still performed acceptably, and even comparably, when assessed against established epigenetic aging clocks.

A deeper scrutiny of the MinLinMo-based prediction models revealed that, although none of them consisted of more than 15 explanatory variables, there were still several variables that hardly contributed to the prediction performance; the aging clock- and birth weight predictors only needed 4 variables while the gestational age clock needed 8 for comparable prediction performance to the full MinLinMo models.

The two strongest predictors of age were CpGs associated with the *ELOVL2* and *KLF14* genes ($R^2=0.82$ for both) that are linked to metabolism [27, 28]. Previously it was reported that caloric restriction may influence DNAm in mammals [44]. Moreover, it has recently been shown that DNAm also scales with longevity in mammals [45]. Hence, the MinLinMo aging clock may thus be driven by constraints related to metabolism. If not directly [46], then perhaps indirectly. The last two predictors of the FCC were linked to the *DNTY* gene, which has no known function, and the *RNF180* gene which has been identified as a tumour suppressor [29]. These CpGs only explained a small fraction of the variance compared to the CpGs linked to the metabolism genes (see Additional file 2). An additional CpG, linked to the gene *FHL2*, also associated with aging [47, 48], was selected by MinLinMo (see Additional files 2 and 4) but not included in the FCC. The reason was due to the negligible model improvement in terms of $R^2$ suggesting that the CpG related to *FHL2* is strongly associated with the other CpGs in the FCC.

In terms of DNAm, far less is known about gestational age than age. However, some similarities have been observed. For instance, as previously noted, CpGs linked to the *ELOVL2* gene appears to explain a substantial part of the variance related to aging [48]. For gestational age, the same has been noted for the *NCOR2* gene [49, 50]. Not only has *NCOR2* been linked to development [9], but so has 3 other genes linked to CpGs in the ECC, namely: *IGF2BP1*, *SETBP1* and *SRC* [9, 30–32], with $R^2=0.54$ compared to $R^2=0.63$ for the full model (see Additional file 2). Hence, as the MinLinMo aging clock appears to be linking age with metabolism, the MinLinMo gestational age clock associates gestational age with development, at least to some extent as measured from cord blood. Less variance was explained by the gestational age clock than for the age clock, with regards to all methods, suggesting additional, but unidentified, mechanisms may be involved, in addition to the ones already mentioned above.

The least accurate predictor, in terms of those tested here, was the birth weight predictor with an $R^2<0.5$ for all methods. Although a relation between DNAm and birth weight has been known for some time [37] this is, to the best of our knowledge, the first DNAm based birth weight predictor. While MinLinMo selected 10 predictors in total for the birth weight predictor, it was found that most of the variance ($R^2=0.4$, see Additional file 2 for more details) could be explained by gestational age and 3 CpGs related to genes involved with metabolism, i.e. *NEDD4L*, *LOC400867*, *FOXO3* [39–41]. Interestingly, the *FOXO3* gene has also been linked to life span [51]. By far, the strongest predictor for birth weight was gestational age ($R^2=0.32$ of $R^2=0.41$) indicating that separating predictors related to gestational age from birth weight could be challenging.

All of the prediction models generated here exhibited a striking degree of redundancy. This was immediately obvious for the Lasso models where the 1SE penalty reduced the number of predictors by many hundreds, hardly affecting prediction accuracy. Redundancy was also observed with MinLinMo; an age predictor with adjusted parameters, Pearson correlation set to 0.005, $\Delta R^2 =0.005$ and the residual correlation to 0.05, only increased the model precision with $\Delta R^2=0.01$ (i.e. the model explained 1% more of the variance), at the cost of adding 12 predictors to the 15 predictor model (i.e. 27 CpGs in total). Even more surprising was the fact that considerable redundancy was also discovered with the smaller MinLinMo clocks as demonstrated with the FCC, ECC and the reduced birth weight predictor (see Additional file 2). It is tempting to speculate that this redundancy is related to the nature of DNAm in the human genome (methylomes) and the epigenetic arrays responsible for the datasets used to build and test the prediction models described in this study. These arrays measure DNAm at CpG di-nucleotides in the human genome [8]. There are approximately 28 million CpGs in the human genome [8], the majority of which, maybe up to 80% [52], have a determined methylation status in the form of being methylated. That leaves approximately 5.6 million CpGs with a variable methylation status. The EPIC array contains roughly 1/7 of these CpG sites and since human methylomes, at least as measured using DNAm arrays, are highly correlated [22], it is possible that the observed redundancy is due to CpGs correlating with other CpGs, present or not on the DNAm array.

### Drawbacks

While MinLinMo is able to produce fairly accurate models fast, it does so at a cost. One drawback is that it can only work with linear models, which may not always be appropriate. For instance, a recent epigenetic age clock manages to outperform large linear models with only 30 CpGs by employing non-linear methodology [53]. Another disadvantage with MinLinMo is that the prediction models tend to be less accurate than the larger models produced by the Elastic Net. Of course, if increased understanding of what a prediction model actually emphasizes is important, a loss of 5–10% prediction accuracy may be justifiable for a model with a high $R^2$ and only a fraction of the number of explanatory variables. If prediction accuracy is the goal, regardless of the number of predictors included in the model, then the Elastic Net is likely a better choice than MinLinMo which also becomes less efficient when the model size increases. An additional potential problem with MinLinMo is the way the predictors are selected using a priority queue. With many predictors correlating similarly with the outcome, MinLinMo can potentially miss out on predictors resulting from complicated correlation structures. Finally, MinLinMo may not be optimal in terms of rejecting spuriously correlating variables, at least when compared to the Lasso with 1SE rule penalties, but is in this regard subjected to the same limitations as ordinary least squares regression.

## Conclusion

MinLinMo is presented as a tool for creating smaller and more parsimonious prediction models with acceptable accuracy to larger ones such as the Elastic Net. Examples were presented for epigenetic adult- and gestational age as well as birth weight.

Unlike most prediction models that prioritize increasing accuracy, MinLinMo uniquely emphasizes creating parsimonious and comprehensible models, potentially enhancing clinical utility. By assaying CpGs selected by MinLinMo from a newborn’s blood, both the expected time from conception and corresponding birth weight can be estimated. Deviations from these expected values could indicate adverse health outcomes. Moreover, by interrogating the methylation status of just a handful of CpGs from a drop of blood, the age of an individual can be determined to within a few years.

Not only is MinLinMo fast, but it strives to keep a low memory foot-print allowing it to run large $n\ll p$ datasets on desktop computers and even laptops. Although designed with large datasets in mind, MinLinMo performs equally well as a variable selector on small datasets where the focus is to avoid indirectly, or loosely correlated variables and produce as small models as justifiable. As such, MinLinMo could also be useful for polygenic risk score estimation.

Finally, we found that the Lasso/Elastic Net prediction models described here reduced the number of selected variables considerably when the “one standard error rule” penalty was employed, instead of the minimum MSE penalty, with only negligible loss in accuracy.

## Supplementary information

Additional file 1: Compilation, installation and usage of MinLinMo for Linux, OS X and Windows platforms

Additional file 2: Statistical results from model comparisons in Excel format

Additional file 3: Outline of quality control of DNAm datasets used to test and validate MinLinMo and Lasso models

Additional file 4: Predictors/CpGs selected available on test dataset as well as estimated coefficients and corresponding information for MinLinMo and Elastic Net adult age models in Excel format

Additional file 5: Distribution density plots of residuals from given outcomes regressed on predicted 

Additional file 6: Overview of overlapping predictors/CpGs between published/Elastic Net and Min- LinMo generated prediction models

Additional file 7: Predictors/CpGs selected, together with estimated coefficients, and corresponding information for MinLinMo and Elastic Net gestational age models in Excel format

Additional file 8: Predictors/CpGs selected and corresponding information as well as coefficient estimates for MinLinMo and Elastic Net birth weight models in Excel format

## Data Availability

Project name: MinLinMo Home page:https: //github.com/JonBohlin/MinLinMo Operating system(s): Windows, OS X, Linux, Raspberry Pi OS Programming language: C++ version 14 Other requirements: GNU scientific library License: GPL 3.0 Any restrictions to use by non-academics: None. Access to the analysed DNA methylation datasets can be obtained by applying to the Norwegian Institute of Public Health (NIPH). Restrictions apply regarding the availability of these data, which were originally used under specific approvals for the current study and are therefore not publicly available. Access can only be given after approval by REK under the provision that the applications are consistent with the consent provided. An application form can be found on the NIPH website at https://www.fhi.no/en/studies/moba/. Specific questions regarding access to data in this study can also be directed to Siri E. Håberg (Siri.Haberg@fhi.no). The dataset GSE116339, used for testing the adult age predictor, can be downloaded from from the Gene Expression Omnibus web-site: https://www.ncbi.nlm.nih.gov/geo/. No data used in the manuscript was generated specifically for this study.

## Abbreviations

1SE: One standard error within minimum prediction error
1SE rule: $\lambda$ 1SE within minimum MSE for more parsimonious models
$\alpha$: Shrinkage parameter, decides Elastic Net model
BLAS: Basic linear algebra subsystem, library for basic linear algebra procedures
CpG: DNA methylation site (CG dinucleotide)
csv: Comma-separated files
$\Delta R^2$: Difference in variance explained
DNAm: DNA methylation
DoF: Degrees of freedom
ECC Eight: CpG clock, a small gestational age predictor consisting of 8 predictors
${F_{lower}}$: Lower 95% confidence interval for F statistic
${F_{upper}}$: Upper 95% confidence interval for F statistic
${\#(F<F_{lower})}$: Number of F statistics below 95% confidence interval
${\#(F<F_{upper})}$: Number of F statistics above 95% confidence interval
FCC: Four CpG clock, a small epigenetic age predictor consisting of 4 predictors
glmnet: R-package for estimating elastic net models
GSL: GNU scientific libarary, state-of-the-art algorithms for scientific computing
${\lambda }$: Elastic net MSE penalty parameter
Lasso 1SE: Lasso model with 1SE MSE penalty with an emphasis on parsimony
Lasso Min: Lasso model with minimum MSE penalty with an emphasis on prediction accuracy
MoBa: The Norwegian mother, father and child cohort study
MSE: Mean square error, a measure of prediction error
${n\ll p}$: Datasets with considerably more predictors than samples
${R^2}$: Goodness-of-fit statistic for linear models (% variance explained)
RAM: Random access memory, available memory for computation
SIMD: Single instruction multiple data, increased parallelization
SNP: Single nucleotide polymorphism, essentially DNA mutation
QC: Quality control, preprocessing of DNA methylation data
QR decomposition: A method for calculation ordinary least squares estimates

## Code availability

Compiled files, a testing data set and source files for MinLinMo, together with instructions for installation and usage, are available at https://github.com/JonBohlin/MinLinMo A compiled version for the Windows platform together with necessary libraries can be found here (doi:10.5281/zenodo.11399859): https://zenodo.org/records/11399859

## References

[1] James G, Witten D, Hastie T, Tibshirani R, et al. An introduction to statistical learning. New York: Springer; 2013.

[2] Breiman L. Statistical modeling: the two cultures (with comments and a rejoinder by the author). Stat Sci. 2001;16(3):199–231.

[3] Hastie T, Tibshirani R, Friedman JH, Friedman JH. The Elements of Statistical Learning: Data Mining, Inference, and Prediction, vol. 2. New York: Springer; 2009.

[4] Meinshausen N, Bühlmann P. Stability selection. J R Stat Soc Ser B Stat Methodol. 2010;72(4):417–73.

[5] Zou H, Hastie T. Regularization and variable selection via the elastic net. J R Stat Soc Ser B Stat Methodol. 2005;67(2):301–20.

[6] Engebretsen S, Bohlin J. Statistical predictions with glmnet Clinical epigenetics. 2019;11(1):1–3. 10.1186/s13148-019-0730-1 PMC6708235 31443682

[7] Tibshirani R. Regression shrinkage and selection via the lasso. J R Stat Soc Ser B Stat Methodol. 1996;58(1):267–88.

[8] Pidsley R, Zotenko E, Peters TJ, Lawrence MG, Risbridger GP, Molloy P, Van Djik S, Muhlhausler B, Stirzaker C, Clark SJ. Critical evaluation of the illumina methylationepic beadchip microarray for whole-genome DNA methylation profiling. Genome Biol. 2016;17(1):1–17. 27717381 10.1186/s13059-016-1066-1 PMC5055731

[9] Haftorn KL, Romanowska J, Lee Y, Page CM, Magnus PM, Håberg SE, Bohlin J, Jugessur A, Denault WR. Stability selection enhances feature selection and enables accurate prediction of gestational age using only five DNA methylation sites. Clin Epigenetics. 2023;15(1):114. 37443060 10.1186/s13148-023-01528-3 PMC10339624

[10] Edwards JR, Yarychkivska O, Boulard M, Bestor TH. DNA methylation and DNA methyltransferases. Epigenetics & chromatin. 2017;10(1):1–10. 28503201 10.1186/s13072-017-0130-8 PMC5422929

[11] Friedman J, Hastie T, Tibshirani R. Regularization paths for generalized linear models via coordinate descent. J Stat Softw. 2010;33(1):1. 20808728 PMC2929880

[12] R Core Team: R: A Language and Environment for Statistical Computing. R foundation for statistical computing, Vienna, Austria (2021). R Foundation for Statistical Computing. https://www.R-project.org/

[13] Gough B GNU scientific library reference manual. Network Theory Ltd., * (2009)

[14] Team TG GNU scientific library (2023). https://www.gnu.org/software/gsl/

[15] Householder AS. Unitary triangularization of a nonsymmetric matrix. J ACM (JACM). 1958;5(4):339–42.

[16] Magnus P, Birke C, Vejrup K, Haugan A, Alsaker E, Daltveit AK, Handal M, Haugen M, Høiseth G, Knudsen GP, et al. Cohort profile update: the norwegian mother and child cohort study (MoBa). Int J Epidemiol. 2016;45(2):382–8. 27063603 10.1093/ije/dyw029

[17] Analytics R, Weston S doMC: Foreach Parallel Adaptor for ’parallel’. (2022). R package version 1.3.8. https://CRAN.R-project.org/package=doMC

[18] Hannum G, Guinney J, Zhao L, Zhang L, Hughes G, Sadda S, Klotzle B, Bibikova M, Fan J-B, Gao Y, et al. Genome-wide methylation profiles reveal quantitative views of human aging rates. Mol Cell. 2013;49(2):359–67. 23177740 10.1016/j.molcel.2012.10.016 PMC3780611

[19] Horvath S. DNA methylation age of human tissues and cell types. Genome Biol. 2013;14(10):1–20. 10.1186/gb-2013-14-10-r115 PMC4015143 24138928

[20] Horvath S, Oshima J, Martin GM, Lu AT, Quach A, Cohen H, Felton S, Matsuyama M, Lowe D, Kabacik S, et al. Epigenetic clock for skin and blood cells applied to hutchinson gilford progeria syndrome and ex vivo studies. Aging (Albany NY). 2018;10(7):1758. 30048243 10.18632/aging.101508 PMC6075434

[21] Pawitan Y. In all likelihood: statistical modelling and inference using likelihood. Oxford: Oxford University Press; 2001.

[22] Nustad HE, Steinsland I, Ollikainen M, Cazaly E, Kaprio J, Benjamini Y, Gervin K, Lyle R. Modeling dependency structures in 450k DNA methylation data. Bioinformatics. 2022;38(4):885–91. 34788815 10.1093/bioinformatics/btab774 PMC8796368

[23] Davison AC, Hinkley DV. Bootstrap methods and their application, vol. 1. New York: Cambridge University Press; 1997.

[24] Lee Y, Haftorn KL, Denault WR, Nustad HE, Page CM, Lyle R, Lee-Ødegård S, Moen G-H, Prasad RB, Groop LC, et al. Blood-based epigenetic estimators of chronological age in human adults using DNA methylation data from the illumina methylationepic array. BMC Genomics. 2020;21:1–13. 10.1186/s12864-020-07168-8 PMC7590728 33109080

[25] Curtis SW, Cobb DO, Kilaru V, Terrell ML, Kennedy EM, Marder ME, Barr DB, Marsit CJ, Marcus M, Conneely KN, et al. Exposure to polybrominated biphenyl (PBB) associates with genome-wide DNA methylation differences in peripheral blood. Epigenetics. 2019;14(1):52–66. 30676242 10.1080/15592294.2019.1565590 PMC6380401

[26] Efron B, Hastie T, Tibshirani R. Discussion: the dantzig selector: Statistical estimation when p is much larger than n. Ann Stat. 2007;35(6):2358–64.

[27] Zhu T, Zheng SC, Paul DS, Horvath S, Teschendorff AE. Cell and tissue type independent age-associated DNA methylation changes are not rare but common. Aging (Albany NY). 2018;10(11):3541. 30482885 10.18632/aging.101666 PMC6286821

[28] Slieker RC, Relton CL, Gaunt TR, Slagboom PE, Heijmans BT. Age-related DNA methylation changes are tissue-specific with ELOVL2 promoter methylation as exception. Epigenetics & chromatin. 2018;11:1–11. 29848354 10.1186/s13072-018-0191-3 PMC5975493

[29] Wu Z, Liu H, Sun W, Du Y, He W, Guo S, Chen L, Zhao Z, Wang P, Liang H, et al. RNF180 mediates STAT3 activity by regulating the expression of RhoC via the proteasomal pathway in gastric cancer cells. Cell Death & Disease. 2020;11(10):881. 33082325 10.1038/s41419-020-03096-3 PMC7575565

[30] Núñez L, Buxbaum AR, Katz ZB, Lopez-Jones M, Nwokafor C, Czaplinski K, Pan F, Rosenberg J, Monday HR, Singer RH. Tagged actin mRNA dysregulation in IGF2BP1 -/- mice. Proc Natl Acad Sci. 2022;119(37):2208465119. 10.1073/pnas.2208465119 PMC9477413 36067310

[31] Piazza R, Magistroni V, Redaelli S, Mauri M, Massimino L, Sessa A, Peronaci M, Lalowski M, Soliymani R, Mezzatesta C, et al. SETBP1 induces transcription of a network of development genes by acting as an epigenetic hub. Nat Commun. 2018;9(1):2192. 29875417 10.1038/s41467-018-04462-8 PMC5989213

[32] Ortiz MA, Mikhailova T, Li X, Porter BA, Bah A, Kotula L. Src family kinases, adaptor proteins and the actin cytoskeleton in epithelial-to-mesenchymal transition. Cell Commun Signal. 2021;19(1):67. 34193161 10.1186/s12964-021-00750-x PMC8247114

[33] Mangodt T, Vanden Driessche K, Norga K, Moes N, De Bruyne M, Haerynck F, Bordon V, Jansen A, Jonckheere A. Central nervous system manifestations of LRBA deficiency: case report of two siblings and literature review. BMC Pediatr. 2023;23(1):353. 37443020 10.1186/s12887-023-04182-z PMC10339488

[34] Fling SP, Arp B, Pious D. HLA-DMA and -DMB genes are both required for MHC class II/peptide complex formation in antigen-presenting cells. Nature. 1994;368(6471):554–8. 8139690 10.1038/368554a0

[35] Bojjireddy N, Guzman-Hernandez ML, Reinhard NR, Jovic M, Balla T. EFR3s are palmitoylated plasma membrane proteins that control responsiveness to G-protein-coupled receptors. J Cell Sci. 2015;128(1):118–28. 25380825 10.1242/jcs.157495 PMC4282049

[36] Xu J, Jiang J, Yin C, Wang Y, Shi B. Identification of ATP6V0A4 as a potential biomarker in renal cell carcinoma using integrated bioinformatics analysis. Oncol Lett. 2023;26(3):1–12. 10.3892/ol.2023.13952 PMC10407721 37559594

[37] Engel SM, Joubert BR, Wu MC, Olshan AF, Håberg SE, Ueland PM, Nystad W, Nilsen RM, Vollset SE, Peddada SD, et al. Neonatal genome-wide methylation patterns in relation to birth weight in the norwegian mother and child cohort. Am J Epidemiol. 2014;179(7):834–42. 24561991 10.1093/aje/kwt433 PMC3969535

[38] Küpers LK, Monnereau C, Sharp GC, Yousefi P, Salas LA, Ghantous A, Page CM, Reese SE, Wilcox AJ, Czamara D, et al. Meta-analysis of epigenome-wide association studies in neonates reveals widespread differential DNA methylation associated with birthweight. Nat Commun. 2019;10(1):1893. 31015461 10.1038/s41467-019-09671-3 PMC6478731

[39] Li M, Sun G, Wang P, Wang W, Cao K, Song C, Sun Y, Zhang Y, Zhang N. Research progress of Nedd4L in cardiovascular diseases. Cell Death Discovery. 2022;8(1):206. 35429991 10.1038/s41420-022-01017-1 PMC9013375

[40] Agarwal T, Lyngdoh T, Khadgawat R, Prabhakaran D, Chandak GR, Walia GK. Genetic architecture of adiposity measures among Asians: findings from GWAS. Ann Hum Genet. 2023;87(6):255–73. 37671428 10.1111/ahg.12526

[41] Chen R, Morris BJ, Donlon TA, Masaki KH, Willcox DC, Davy PM, Allsopp RC, Willcox BJ. FOXO3 longevity genotype mitigates the increased mortality risk in men with a cardiometabolic disease. Aging (Albany NY). 2020;12(23):23509. 33260156 10.18632/aging.202175 PMC7762472

[42] Maié T, Schmidt M, Erz M, Wagner W, Costa IG. CimpleG: finding simple CpG methylation signatures. Genome Biol. 2023;24(1):161. 37430364 10.1186/s13059-023-03000-0 PMC10332104

[43] Koestler DC, Jones MJ, Usset J, Christensen BC, Butler RA, Kobor MS, Wiencke JK, Kelsey KT. Improving cell mixture deconvolution by identifying optimal DNA methylation libraries (idol). BMC Bioinf. 2016;17:1–21. 10.1186/s12859-016-0943-7 PMC4782368 26956433

[44] Maegawa S, Lu Y, Tahara T, Lee JT, Madzo J, Liang S, Jelinek J, Colman RJ, Issa J-PJ. Caloric restriction delays age-related methylation drift. Nat Commun. 2017;8(1):539. 28912502 10.1038/s41467-017-00607-3 PMC5599616

[45] Crofts SJ, Latorre-Crespo E, Chandra T. DNA methylation rates scale with maximum lifespan across mammals. Nature Aging. 2024;4(1):27–32. 38049585 10.1038/s43587-023-00535-6 PMC10798888

[46] Hatton IA, Dobson AP, Storch D, Galbraith ED, Loreau M. Linking scaling laws across eukaryotes. Proc Natl Acad Sci. 2019;116(43):21616–22. 31591216 10.1073/pnas.1900492116 PMC6815163

[47] Bacalini MG, Deelen J, Pirazzini C, De Cecco M, Giuliani C, Lanzarini C, Ravaioli F, Marasco E, Van Heemst D, Suchiman HED, et al. Systemic age-associated DNA hypermethylation of ELOVL2 gene: in vivo and in vitro evidences of a cell replication process. J Gerontol Ser A: Biomed Sci Med Sci. 2017;72(8):1015–23. 10.1093/gerona/glw185 PMC5861890 27672102

[48] Naue J. Getting the chronological age out of DNA: using insights of age-dependent DNA methylation for forensic DNA applications. Genes & Genomics. 2023;45(10):1239–61. 37253906 10.1007/s13258-023-01392-8 PMC10504122

[49] Bohlin J, Håberg SE, Magnus P, Reese SE, Gjessing HK, Magnus MC, Parr CL, Page C, London SJ, Nystad W. Prediction of gestational age based on genome-wide differentially methylated regions. Genome Biol. 2016;17:1–9. 27717397 10.1186/s13059-016-1063-4 PMC5054559

[50] Haftorn KL, Lee Y, Denault WR, Page CM, Nustad HE, Lyle R, Gjessing HK, Malmberg A, Magnus MC, Næss Ø, et al. An EPIC predictor of gestational age and its application to newborns conceived by assisted reproductive technologies. Clin Epigenetics. 2021;13:1–13. 33875015 10.1186/s13148-021-01055-z PMC8056641

[51] Morris BJ, Willcox DC, Donlon TA, Willcox BJ. Foxo3: a major gene for human longevity-a mini-review. Gerontology. 2015;61(6):515–25. 25832544 10.1159/000375235 PMC5403515

[52] Kaluscha S, Domcke S, Wirbelauer C, Stadler MB, Durdu S, Burger L, Schübeler D. Evidence that direct inhibition of transcription factor binding is the prevailing mode of gene and repeat repression by DNA methylation. Nat Genet. 2022;54(12):1895–906. 36471082 10.1038/s41588-022-01241-6 PMC9729108

[53] Varshavsky M, Harari G, Glaser B, Dor Y, Shemer R. Kaplan T Accurate age prediction from blood using a small set of DNA methylation sites and a cohort-based machine learning algorithm. Cell Rep Methods. 2023;3(9): 100567. 37751697 10.1016/j.crmeth.2023.100567 PMC10545910

