# Supplementary material for: MinLinMo: a minimalist approach to variable selection and linear model prediction
Source: BMC Bioinformatics. 2024 Dec 18;25:380. doi: 10.1186/s12859-024-06000-4 (PMC11654326; doi:10.1186/s12859-024-06000-4)
Supplement: Supplementary file 1 — Additional file 1: Compilation, installation and usage of MinLinMo for Linux, OS X and Windows platforms [file 12859_2024_6000_MOESM1_ESM.pdf]

Compiling and installing MinLinMo

MinLinMo was written in C++ version 14. It has been developed for both Intel and ARM processors. For Intel processors both multi-threading and AVX2 are employed for maximum performance. For ARM processors, NEON is used instead. As AVX2 supports 256 bit registers parts of MinLinMo will likely run faster on Intel processors. During the model building phase, which is also the most time consuming phase, multi-threading was not found to improve performance.

To compile MinLinMo for Ubuntu-type Linux make sure that the GNU Scientific Library (GSL) is installed. This can be installed with:
sudo apt install libgsl-dev
To compile:
g++ MinLinMo.cpp -march=core-avx2 -lgsl -lblas -lpthread -O3 -o MinLinMo -std=c++14

For Apple silicon, use Homebrew to install GSL:
brew install gsl

For the ARM (Apple silicon) OS X version use:
g++ MinLinMo.cpp -lpthread -std=c++14 -O3 -lgsl -lblas -I /opt/homebrew/Cellar/gsl/2.7.1/include/ -L /opt/homebrew/Cellar/gsl/2.7.1/lib/ -march=armv8-a -o MinLinMo

For ARM Linux the following should hopefully work (but has not been tested):
g++ MinLinMo.cpp -march=armv8-a -lgsl -lblas -lpthread -O3 -o MinLinMo -std=c++14

For Intel OS X this will hopefully work:
g++ MinLinMo.cpp -lpthread -std=c++14 -O3 -lgsl -lblas -I /opt/homebrew/Cellar/gsl/2.7.1/include/ -L /opt/homebrew/Cellar/gsl/2.7.1/lib/ -march= core-avx2

For Windows it likely easiest to install GSL through Visual Studio 2017 or later. This can be done as follows:

- Create a new C++ project, right click the project name under Solution Explorer and choose "Manage Nuget Packages"
- Click the Browser tab and search with the keywords "Microsoft.gsl", which will filter out the Microsoft GSL version. It can now be installed by clicking the install button

MinLinMo can now be compiled with the Microsoft C++ compiler from Visual Studio.

If there are no errors, type:

./MinLinMo -h

in the directory containing MinLinMo for Linux or OS X to get an overview of MinLinMo's command line arguments. For the Windows version type

MinLinMo.exe -h

To test MinLinMo on data, create a random data set, either with Python:

```python
import numpy as np
import pandas as pd
numcols = 50000
numrows = 1000
matrix = np.random.normal(0, 1, (numrows, numcols))
headers = ["V" + str(i) for i in range(0, numcols)]
df = pd.DataFrame( matrix, columns=headers)
df.to_csv("random_matrix.csv", index=False)

# Outcome vector
vector = np.random.normal(0,1,numrows)
header = ["Outcome"]
outc = pd.DataFrame(vector, columns=header)
outc.to_csv("Outcome.csv", index=False)
```

or R:

```r
library(data.table)
numcols <- 50000
numrows <- 1000
matr <- matrix( rnorm( numcols * numrows ), ncol=numcols, nrow=numrows) # nolint
headers <- paste("pred",1:numcols, sep="") # nolint
colnames(matr) <- headers
df <- as.data.frame( matr ) # nolint
fwrite(matr, file="random_matrix.csv", quote=F) # nolint

vect <- rnorm( numrows ) # nolint
outc <- as.data.frame( vect ) # nolint
names(outc) <- "Outcome"
fwrite(outc, file="Outcome.csv", quote=F) # nolint
```

MinLinMo can now be tested with the generated data by typing:

./MinLinMo -y Outcome.csv -X random_matrix.csv

where -y is the command line option for the outcome vector in file Outcome.csv and -X is the command line argument for the predictor matrix file (random_matrix.csv).

Using MinLinMo to predict

To select variables, MinLinMo creates a linear model. The estimated coefficients of this model can be used to predict the outcome on a test data set having predictors with the same names as MinLinMo selected. To do this, add the -O option to MinLinMo together with a file name:

./MinLinMo -y Outcome.csv -X random_matrix.csv -O ests.txt

The file ests.txt contains the outputted MinLinMo estimates (column named “est”) together with the corresponding predictor names (column named “id”). These estimates can now be loaded into R with:

ests <- read.table(“ests.txt”, header=T)

If you type “ests” now you should see something like:

```
       est        id
1 25.19290 intercept
2  2.33955    pred19
3  1.55191    pred38
4  1.78317    pred22
5  1.93599    pred21
```

```
6 -1.26850     pred4
```

Load the dataset having predictors with corresponding names to the “id” column above:

```
dataset <- read.csv("my_data.csv", header=T)
```

If all MinLinMo selected predictors are present you can now type:

```
ests$id <- as.character(ests$id)
pred <- cbind(1, as.matrix(dataset[,ests$id[-1]])) %*% ests$est
```

pred should now contain the estimated outcome vector, say:

```
pred[1:10]
 [1] 19.42472 26.44910 20.51038 24.96952 25.78452 21.78642 23.25646 24.31260
 [9] 22.68341 20.55097
```
