# Supplementary material for: MinLinMo: a minimalist approach to variable selection and linear model prediction
Source: BMC Bioinformatics. 2024 Dec 18;25:380. doi: 10.1186/s12859-024-06000-4 (PMC11654326; doi:10.1186/s12859-024-06000-4)
Supplement: Supplementary file 3 — Additional file 3: Outline of quality control of DNAm datasets used to test and validate MinLinMo and Lasso models [file 12859_2024_6000_MOESM3_ESM.pdf]

DNA methylation datasets

The DNA methylation (DNAm) data sets used to evaluate MinLinMo were based on the Illumina Infinium MethylationEPIC BeadChip V1.0 array[1], samples taken from either cord blood (newborns) or peripheral blood (adults). For training of the prediction models, both the newborns and the adults were sampled from the Norwegian Mother, father and Child Cohort (MoBa). The datasets and their quality control have been described in previous publications[2-4]. Briefly, samples with more then 10% failing DNAm probes were removed. Those with undetermined sex, assessed using the two first components from a PCA on X chromosome DNAm values, were also removed. The greedycut algorithm from the RnBeads 2.0 package[5] was used to remove samples with outlying DNAm patterns. Remaining samples were corrected for background noise with Enmix.oob[6]. Probes failing the detection p-values test ($p<0.01$) were removed together with all cross-hybridizing DNAm probes. Probes where the 3 last nucleotides overlapped with a single nucleotide polymorphism (SNP) were also removed. BMIQ[7] was used to correct for Type I and Type II probes.

The DNAm data from the newborns consisted of 2,188 samples in total. Of these, 1,750 samples were randomly selected for training the gestational age and birth weight models and the remaining 438 samples were used for testing, each sample consisted of 769,139 probes.

For the adults, the MoBa DNAm dataset used for training contained 1,966 samples in total, each sample consisting of 770,586 DNAm probes.

Testing of the adult age based prediction models was performed on the publicly available dataset GSE116339, also based on peripheral blood, which was downloaded from GEO as a prepared .csv file with QC already performed[9]. The dataset consisted of 678 samples and 763,795 probes after CpGs with missing probes were removed.

[1] Pidsley, R., Zotenko, E., Peters, T. J., Lawrence, M. G., Risbridger, G. P., Molloy, P., ... & Clark, S. J. (2016). Critical evaluation of the Illumina MethylationEPIC BeadChip microarray for whole-genome DNA methylation profiling. Genome biology, 17(1), 1-17.

[2] Magnus, P., Birke, C., Vejrup, K., Haugan, A., Alsaker, E., Daltveit, A. K., ... & Stoltenberg, C. (2016). Cohort profile update: the Norwegian mother and child cohort study (MoBa). *International journal of epidemiology*, *45*(2), 382-388.

[3] Lee, Y., Haftorn, K. L., Denault, W. R., Nustad, H. E., Page, C. M., Lyle, R., ... & Bohlin, J. (2020). Blood-based epigenetic estimators of chronological age in human adults using DNA methylation data from the Illumina MethylationEPIC array. BMC genomics, 21, 1-13.

[4] Håberg, S. E., Page, C. M., Lee, Y., Nustad, H. E., Magnus, M. C., Haftorn, K. L., ... & Lyle, R. (2022). DNA methylation in newborns conceived by assisted reproductive technology. Nature Communications, 13(1), 1896.

[5] Haftorn, K. L., Romanowska, J., Lee, Y., Page, C. M., Magnus, P. M., Håberg, S. E., ... & Denault, W. R. (2023). Stability selection enhances feature selection and enables accurate prediction of gestational age using only five DNA methylation sites. Clinical Epigenetics, 15(1), 114.

[6] Müller, F., Scherer, M., Assenov, Y., Lutsik, P., Walter, J., Lengauer, T., & Bock, C. (2019). RnBeads 2.0: comprehensive analysis of DNA methylation data. Genome biology, 20(1), 1-12.

[7] Xu, Z., Niu, L., Li, L., & Taylor, J. A. (2016). ENmix: a novel background correction method for Illumina HumanMethylation450 BeadChip. Nucleic acids research, 44(3), e20-e20.

[8] Teschendorff, A. E., Marabita, F., Lechner, M., Bartlett, T., Tegner, J., Gomez-Cabrero, D., & Beck, S. (2013). A beta-mixture quantile normalization method for correcting probe design bias in Illumina Infinium 450 k DNA methylation data. Bioinformatics, 29(2), 189-196.

[9] Curtis, S. W., Cobb, D. O., Kilaru, V., Terrell, M. L., Kennedy, E. M., Marder, M. E., ... & Smith, A. K. (2019). Exposure to polybrominated biphenyl (PBB) associates with genome-wide DNA methylation differences in peripheral blood. *Epigenetics*, *14*(1), 52-66.
