# Supplementary material for: MinLinMo: a minimalist approach to variable selection and linear model prediction
Source: BMC Bioinformatics. 2024 Dec 18;25:380. doi: 10.1186/s12859-024-06000-4 (PMC11654326; doi:10.1186/s12859-024-06000-4)
Supplement: Supplementary file 5 — Additional file 5: Distribution density plots of residuals from given outcomes regressed on predicted [file 12859_2024_6000_MOESM5_ESM.pdf]

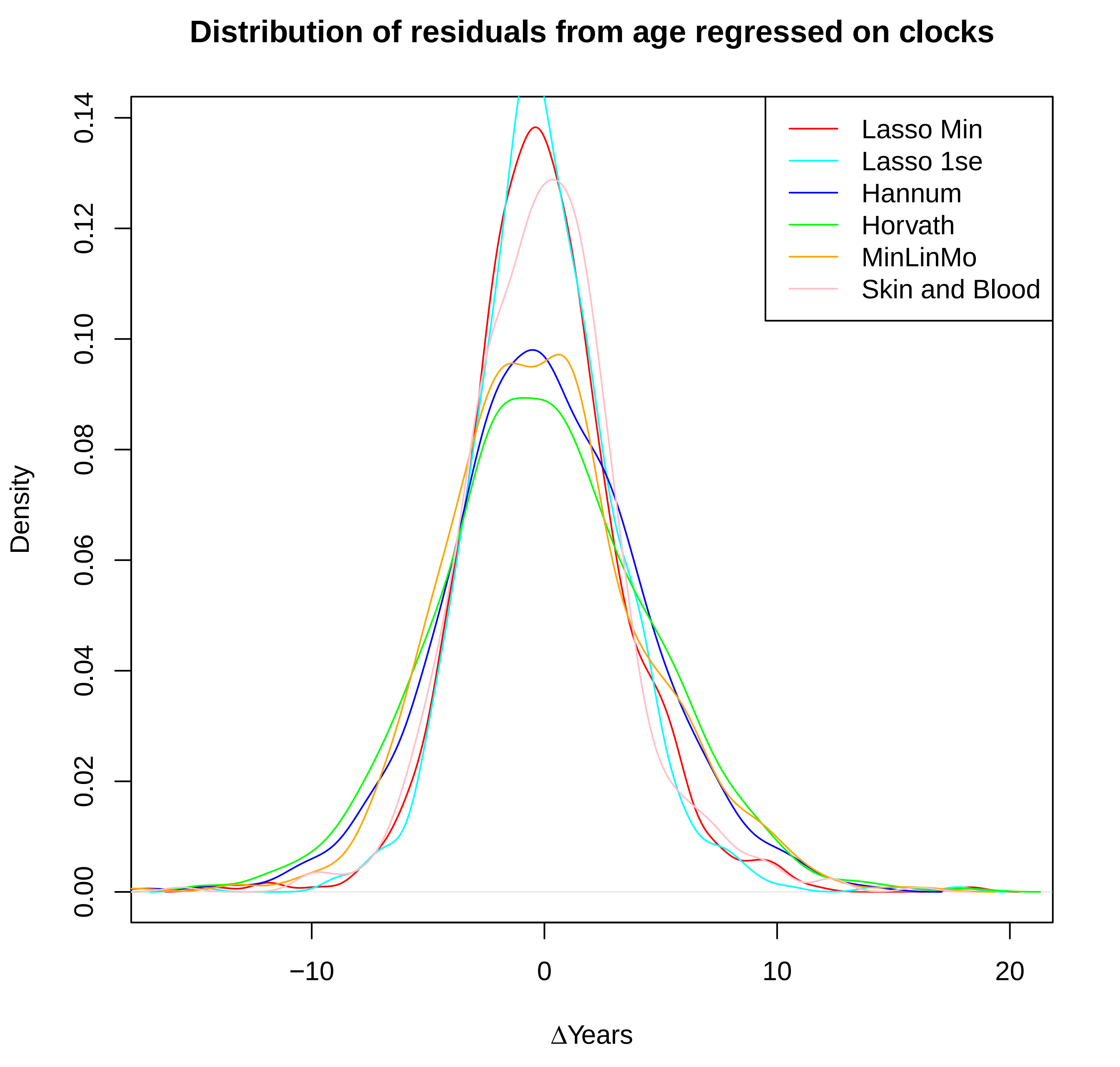

Distribution of residuals from age regressed on clocks
Density
0.00
0.02
0.04
0.06
0.08
0.10
0.12
0.14
−10
0
10
20
ΔYears
Lasso Min
Lasso 1se
Hannum
Horvath
MinLinMo
Skin and Blood

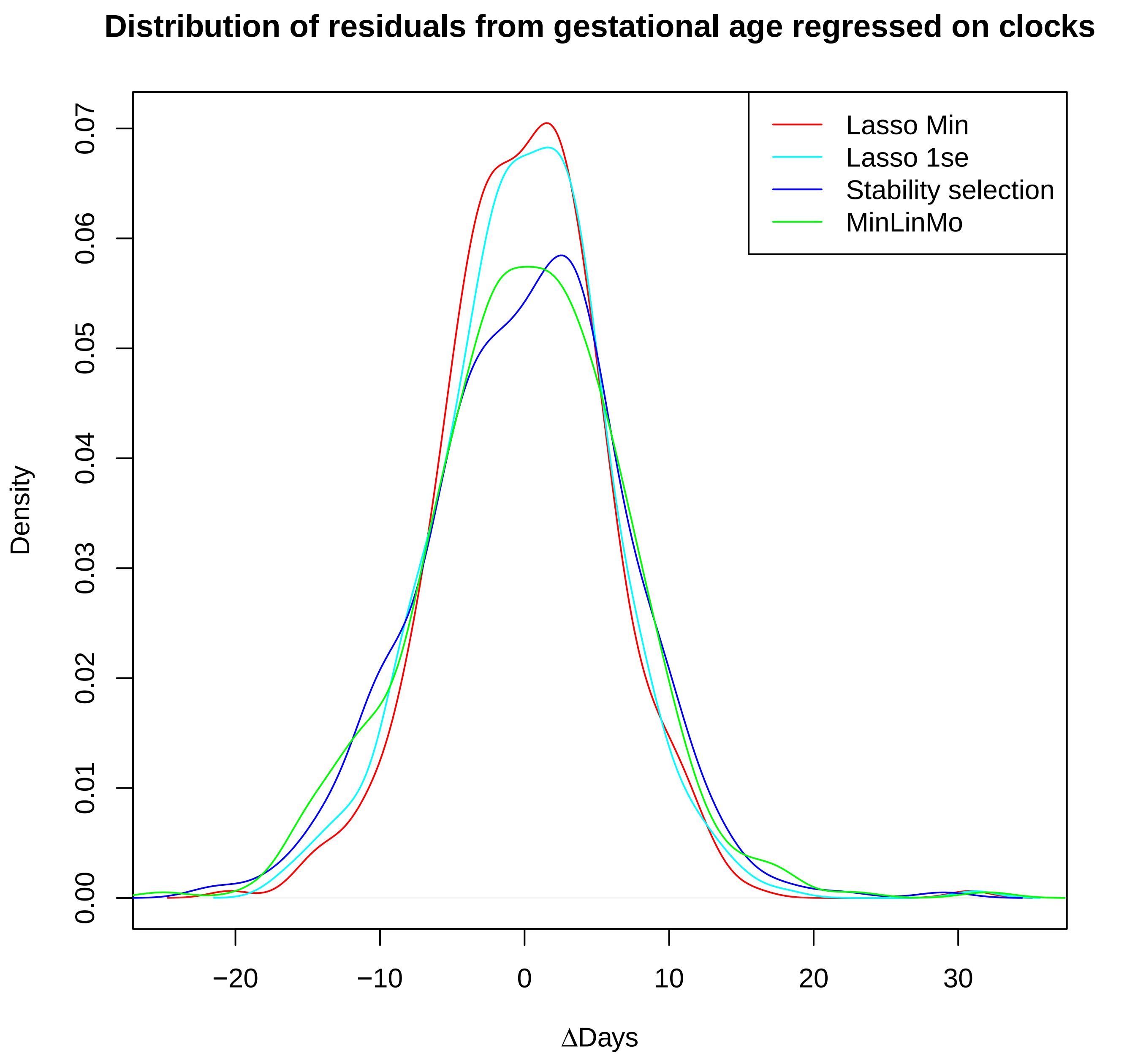

Distribution of residuals from gestational age regressed on clocks
Lasso Min
Lasso 1se
Stability selection
MinLinMo
Density
0.00
0.01
0.02
0.03
0.04
0.05
0.06
0.07
−20
−10
0
10
20
30
ΔDays

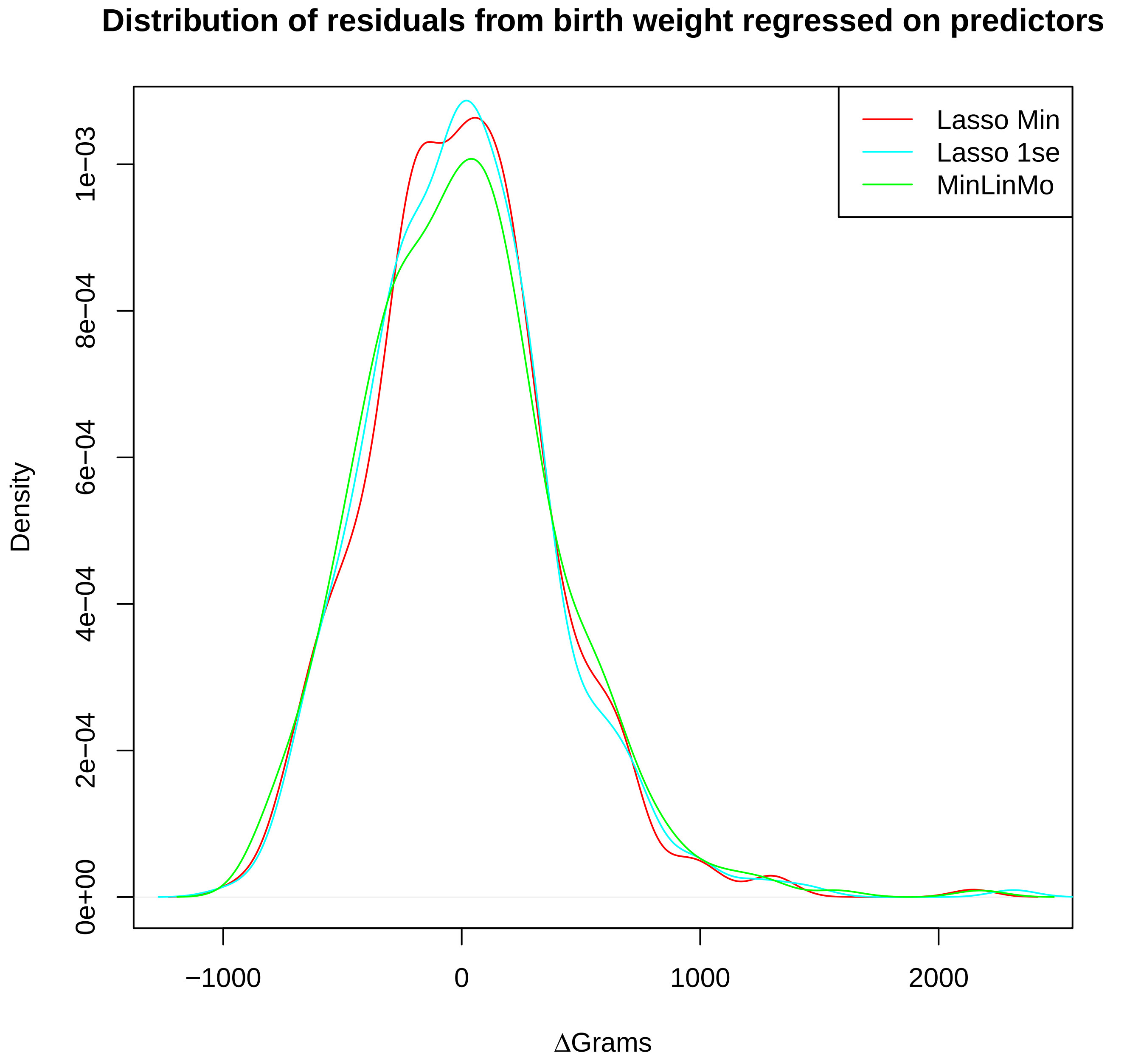

Distribution of residuals from birth weight regressed on predictors
Lasso Min
Lasso 1se
MinLinMo
Density
0e+00
2e−04
4e−04
6e−04
8e−04
1e−03
−1000
0
1000
2000
ΔGrams
