# Supplementary material for: MinLinMo: a minimalist approach to variable selection and linear model prediction
Source: BMC Bioinformatics. 2024 Dec 18;25:380. doi: 10.1186/s12859-024-06000-4 (PMC11654326; doi:10.1186/s12859-024-06000-4)
Supplement: Supplementary file 6 — Additional file 6: Overview of overlapping predictors/CpGs between published/Elastic Net and Min- LinMo generated prediction models [file 12859_2024_6000_MOESM6_ESM.pdf]

Overlap of predictors between different age clocks and MinLinMo

Lasso/Elastic Net based epigenetic age clock min penalty and MinLinMo
"cg10501210" "cg22454769" "cg01620164" "cg03545227" "cg26079664"
"cg17621438" "cg16867657" "cg08097417" "cg13108341"

Lasso/Elastic Net based epigenetic age clock 1SE penalty and MinLinMo
"cg10501210" "cg22454769" "cg01620164" "cg03545227" "cg26079664"
"cg17621438" "cg16867657" "cg08097417" "cg13108341"

Overlap between Hannum[1] clock and MinLinMo
"cg16867657" "cg08097417" "cg22454769" "cg10501210"
Overlap between Horvath[2] Pan-Tissue clock and MinLinMo
None
Overlap between Horvath[3] Skin and Blood clock and MinLinMo
"cg16867657" "cg08097417" "cg22454769" "cg10501210" "cg01620164"

[1]Hannum, G., Guinney, J., Zhao, L., Zhang, L. I., Hughes, G., Sadda, S., ... & Zhang, K. (2013). Genome-wide methylation profiles reveal quantitative views of human aging rates. *Molecular cell*, *49*(2), 359-367.

[2]Horvath, S. (2013). DNA methylation age of human tissues and cell types. *Genome biology*, *14*(10), 1-20.

[3]Horvath, S., Oshima, J., Martin, G. M., Lu, A. T., Quach, A., Cohen, H., ... & Raj, K. (2018). Epigenetic clock for skin and blood cells applied to Hutchinson Gilford Progeria Syndrome and ex vivo studies. *Aging (Albany NY)*, *10*(7), 1758.

Overlap of predictors between different gestational age clocks and MinMoLin

Elastic Net based epigenetic age clock min penalty and MinLinMo

"cg12434132" "cg07749613" "cg22324029" "cg21048168" "cg25975961" "cg11387576" "cg04347477" "cg18183624" "cg21180953" "cg15636859"

Elastic Net based epigenetic age clock 1se penalty and MinLinMo

"cg12434132" "cg07749613" "cg22324029" "cg21048168" "cg25975961" "cg11387576" "cg04347477" "cg18183624" "cg21180953" "cg15636859"

Overlap between Knight[1] clock and MinLinMo

None

Overlap between Bohlin[2] ultra-sound clock and MinLinMo

None

Overlap between Bohlin[2] last menstruation period (LMP) clock and MinLinMo

"cg04347477" "cg07749613" "cg18183624"

Overlap between Haftorn[3] EPIC clock and MinLinMo

"cg04347477" "cg11387576" "cg12434132" "cg25975961" "cg07749613" "cg21180953" "cg18183624" "cg16364693" "cg22324029" "cg21048168"

Overlap between Haftorn[3] 450K clock and MinLinMo

"cg04347477" "cg07749613" "cg18183624" "cg22324029" "cg21048168"

Overlap between Haftorn[3] Embryo Transfer Date (ETD) clock and MinLinMo

"cg04347477" "cg11387576" "cg25975961" "cg07749613" "cg21180953"

Overlap between EPIC[4] EWAS and MinLinMo

"cg04347477" "cg11387576" "cg12434132" "cg25975961" "cg07749613" "cg21180953" "cg18183624" "cg16364693" "cg07904475" "cg02853393" "cg22324029" "cg21048168"

Stability selection clock[5] and MinLinMo

"cg04347477" "cg11387576" "cg25975961" "cg07749613" "cg21180953" "cg18183624"

[1]Knight, A. K., Craig, J. M., Theda, C., Baekvad-Hansen, M., Bybjerg-Grauholm, J., Hansen, C. S., ... & Smith, A. K. (2016). An epigenetic clock for gestational age at birth based on blood methylation data. *Genome biology*, *17*(1), 1-11.

[2]Bohlin, J., Håberg, S. E., Magnus, P., Reese, S. E., Gjessing, H. K., Magnus, M. C., ... & Nystad, W. (2016). Prediction of gestational age based on genome-wide differentially methylated regions. *Genome biology*, *17*(1), 1-9.

[3]Haftorn, K. L., Lee, Y., Denault, W. R., Page, C. M., Nustad, H. E., Lyle, R., ... & Bohlin, J. (2021). An EPIC predictor of gestational age and its application to newborns conceived by assisted reproductive technologies. *Clinical epigenetics*, *13*(1), 1-13.

[4]Haftorn, K. L., Denault, W. R., Lee, Y., Page, C. M., Romanowska, J., Lyle, R., ... & Jugessur, A. (2023). Nucleated red blood cells explain most of the association between DNA methylation and gestational age. *Communications Biology*, *6*(1), 224.

[5]Haftorn, K. L., Romanowska, J., Lee, Y., Page, C. M., Magnus, P. M., Håberg, S. E., ... & Denault, W. R. (2023). Stability selection enhances feature selection and enables accurate prediction of gestational age using only five DNA methylation sites. *Clinical Epigenetics*, *15*(1), 114.

Overlap of CpGs between birth weight predictors

Overlap between Elastic Net min based birth weight predictor and MinLinMo

"GA" "cg16775629" "cg00654448" "cg17375724" "cg11605821"

Overlap between Elastic Net 1se based birth weight predictor and MinLinMo

"GA" "cg16775629" "cg00654448" "cg17375724" "cg11605821"

Overlap between Engel[1] et al birth weight EWAS and MinLinMo

None

Overlap between Küpers[2] et al et al birth weight Meta-EWAS and MinLinMo

"cg00654448" "cg01301319" "cg10453071"

[1]Engel, S. M., Joubert, B. R., Wu, M. C., Olshan, A. F., Håberg, S. E., Ueland, P. M., ... & London, S. J. (2014). Neonatal genome-wide methylation patterns in relation to birth weight in the Norwegian Mother and Child Cohort. *American journal of epidemiology*, *179*(7), 834-842.

[2]Küpers, L. K., Monnereau, C., Sharp, G. C., Yousefi, P., Salas, L. A., Ghantous, A., ... & Felix, J. F. (2019). Meta-analysis of epigenome-wide association studies in neonates reveals widespread differential DNA methylation associated with birthweight. *Nature communications*, *10*(1), 1893.
